# Heartmate 3 as a bridge to heart transplantation in a patient with congenitally corrected transposition of the great arteries: a case report

**DOI:** 10.1186/s13019-022-01793-y

**Published:** 2022-03-26

**Authors:** Yaron D. Barac, Ben Ben-Avraham, Ashraf Hamdan, Rafael Hirsch, Tuvia Ben-Gal, Danny Aravot

**Affiliations:** 1 The Division of Cardiovascular and Thoracic Surgery, Rabin Medical Center, Petach-Tikva, Israel; 2 The Division of Cardiology, Rabin Medical Center, Petach-Tikva, Israel; 3 Sackler Faculty of Medicine, Tel Aviv University, Tel Aviv, Israel

**Keywords:** LVAD, HM3, CCTGA, HTx, Case report

## Abstract

**Background:**

We report the first use of Heartmate 3 (HM3) in a Congenitally corrected Transposition of the Great Arteries (ccTGA) as a Systemic Ventricular Assist Device (SVAD) to treat HF.

**Case presentation:**

A 55 years old man with a Congenitally corrected Transposition of the Great Arteries (ccTGA) a rare condition in which Heart Failure (HF) is a common presentation in adult life and survival without heart transplantation is hardly an option. Systemic Ventricular Assist Device (SVAD) can be an option if an organ does not become available. We present the first ever implantation of HM3 LVAD (Abbott Inc, Chicago IL) implanted to this patient as a bridge to transplantation, *demonstrating the safety and feasibility of the procedure.* Due to the unique mediastinal configuration, 3D cardiac CT reconstruction should be used for planning the procedure—intra ventricular placement of the inflow as well as mediastinal placemat of the outflow and pump.

**Conclusions:**

This successful first use of HM3 as a SVAD for ccTGA patients, opens a novel treatment option for these patients as a bridge for heart transplant or as definitive treatment.

## Introduction

LVAD’s are implanted in increasing numbers, specifically HM3, shown to be superior to other assist devices due to its reliability and minimal adverse events [1]. In contrast to HF patients in congenital heart disease (CHD) patients, LVAD use is rare. ccTGA has a prevalence of < 0.5% of all congenital heart disease, while Dextrocardia is found in 20% of these patients’ [2], ccTGA has both atrioventricular and ventriculoarterial discordance which provides a normal circulation of blood, but with inverted ventricles; the right pumping the systemic flow and the left being the sub-pulmonary chamber. The pressure loaded RV since birth often succumbs in the 4–5th decade of life [3], causing HF. Herein, we demonstrate for the first time the feasibility of use of HM3 in a ccTGA patient.

## Case summary

A 55-year old male with ccTGA in situs-solitus and mesocardiac with recurrent atrial dysrhythmia undergoing several ablation procedures and implanted with a CRT pacemaker due to AV block, and device closure of two secundum ASD’s; Deteriorated to severe HF with moderate tricuspid regurgitation (morphological right ventricle), moderate pulmonary hypertension and high PVR (preventing heart transplant at this stage) and thus required multiple hospitalizations for inotropic support.

**Fig. 1:**
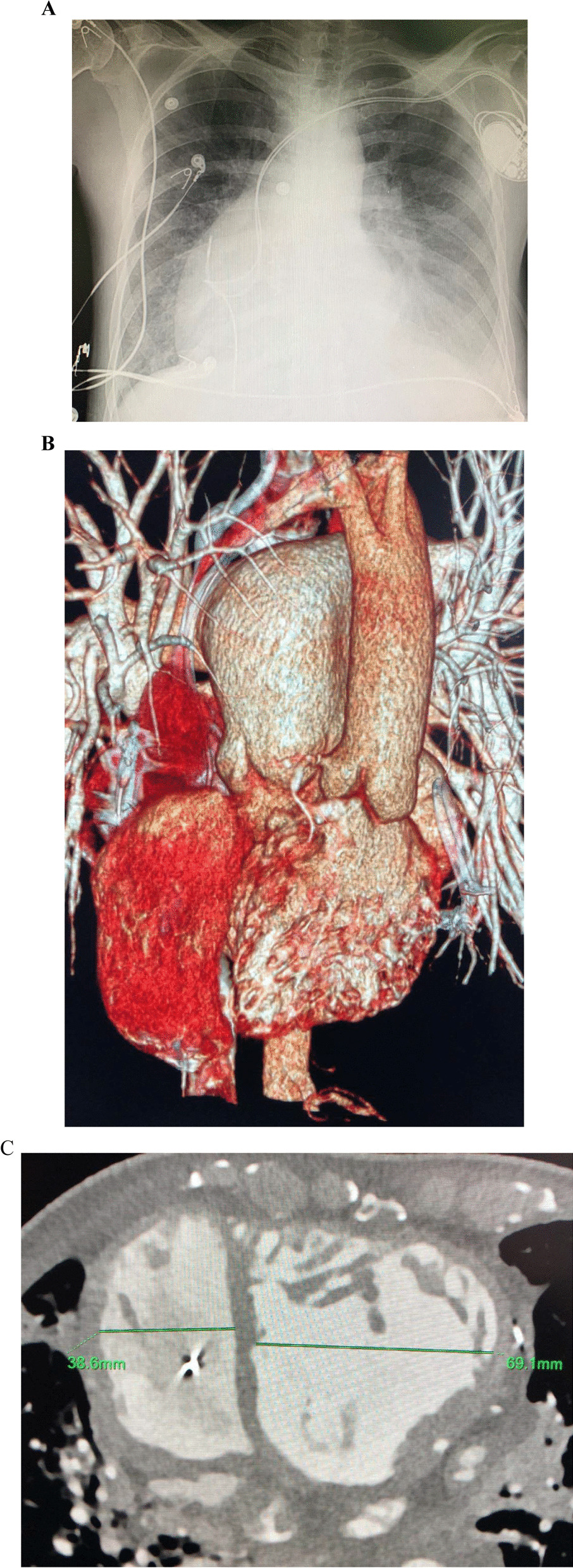
**A** Chest X-Ray of a ccTGA patient with no dextrocardia, the LV can be seen on the right side of the chest. **B** 3D cardiac CT reconstruction of the heart and the great vessels; On the left side a distended PA and on the right side a “thin” aorta. **C** The dimensions of both ventricles are depicted the systemic RV and it’s trabeculation are seen on the left side of the image

**Fig. 2:**
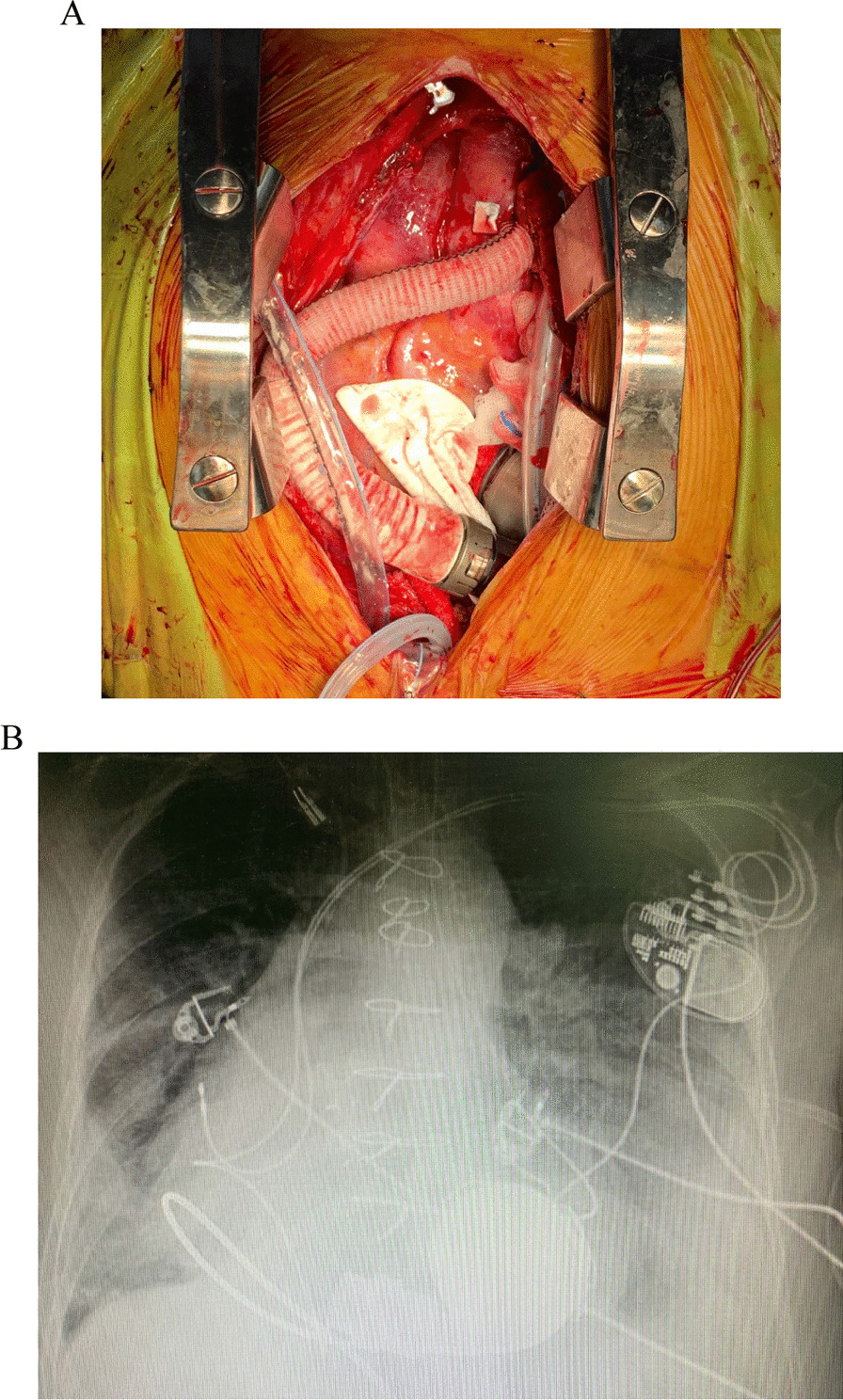
**A** HM3 implanted in the heart of the ccTGA patient, the graft pathway in the chest is unique and longer as the aorta is located left to the PA. **B** Chest X-Ray demonstrating the direction of the inflow cannula in the heart of the ccTGA patient directed anterior to posterior

A HM3 was chosen as a bridge to heart transplant and 3D cardiac CT was performed for verification that the inflow cannula as well as the pump can fit the systemic RV and the chest cavity (Fig. 1); One cannot stress enough the importance of this step to verify the possibility of the HM3 implantation. Several technical challenges have to be addressed e.g. as the ascending aorta is anterior and left to the PA and the systemic morphological RV is significantly trabeculated with a moderator band and papillary muscle adjacent to the inflow cannula planned location. Previous reports have demonstrated that a “regular” placement of the inflow cannula has caused an obstruction by the moderator band [4], thus a proper placement of the inflow cannula in the systemic RV requires adjustment and TEE guidance. Once the hole in the RV is made, the trabeculations should be kept out of the inflow cannula’s way and cut out if needed; One should keep in mind that diaphragmatic placement of the inflow cannula is also an option as previously described [5]. Next, the pump itself is placed through an incision in the pericardium above the phrenic nerve, in the left chest cavity, finally the outflow graft has to “travel” a longer path in the chest due to the location of the aorta (Fig. 2). Hemodynamically the Post-OP of the patient was well, with 5400 RPM and 4.6 L flow as well as good function of the sub-pulmonic ventricle; His hospital course was complicated by a difficulty weaning off the ventilator thus a tracheostomy was made and a gradual weaning course was taken. While the course of this patient in the short and mid-term were good, the long-term results of these patients should still be evaluated in the future.

Thus far very few reports describe the use of LVAD in ccTGA patients, HM2 and lately HVAD were used thus far [6, 7]. HM3 use while waiting for a heart (up to years) is advantageous due to minimal adverse events [1].

## Conclusion

We describe the first use of HM3 in a ccTGA patient thus demonstrating the feasibility of its use as a bridge to heart transplantation; in the future the use of HM3 may be broadened to other congenital indications with complex anatomy. It should be noted that the feasibly of use should be well evaluated pre-op using advanced techniques e.g. 3D cardiac CT reconstruction.

## Data Availability

Yes.

## Abbreviations

HM3: HeartMate 3
LVAD: Left Ventricular Assist Device
CCTGA: Congenitally Corrected Transposition of the Great Arteries
HF: Heart Failure
HTx: Heart Transplantation
SVAD: Systemic Ventricular Assist Device
CHD: Congenital Heart Disease
RV: Right ventricle
ASD: Atrial Septal Defect
TEE: Trans Esophageal Echo
